# Long-term symptoms after SARS-CoV-2 infection in a cohort of hospital employees: duration and predictive factors

**DOI:** 10.1186/s12879-023-08710-1

**Published:** 2024-01-23

**Authors:** Rosalie Gruber, María Verónica Montilva Ludewig, Christina Weßels, Gerlinde Schlang, Svenja Jedhoff, Swetlana Herbrandt, Frauke Mattner

**Affiliations:** 1 Institute of Hygiene, Cologne Merheim Medical Centre, University Hospital of Witten/Herdecke, Ostmerheimer Straße 200, 51109 Köln, Germany; 2 Division of Hygiene and Environmental Medicine, Department of Human Medicine, Faculty of Health, Witten/Herdecke University, Alfred-Herrhausen-Straße 50, 58448 Witten, Germany; 3 Occupational Health Service, Cologne Municipal Hospitals, Neufelder Straße 34, 51067 Köln, Germany; 4 Statistical Consulting and Analysis, Centre for Higher Education, TU Dortmund University, Vogelpothsweg 78, 44227 Dortmund, Germany

**Keywords:** COVID-19, SARS-CoV-2, Post COVID syndrome, Long COVID, Healthcare worker

## Abstract

**Purpose:**

To evaluate the frequency, duration and patterns of long-term coronavirus disease 2019 (COVID-19) symptoms and to analyse risk factors for long-lasting COVID-19 sequelae among a cohort of hospital employees (HEs).

**Methods:**

We conducted a survey regarding persistent COVID-19 related symptoms with all HEs from three medical centres in Cologne, Germany, who were tested SARS-CoV-2 PCR positive from March 2020 until May 2021. Duration of symptoms and possible risk factors for protracted COVID-19 course were analysed.

**Results:**

Of 221 included HEs, a number of 104 HEs (47.1%) reported at least one persisting symptom for more than 90 days after initial SARS-CoV-2 detection. Each one cycle higher initial Ct value significantly increased the chances of overcoming symptoms (odds ratio [OR] 1.05; 95% confidence interval (95%CI) 1.01–1.09; p = 0.019). The occurrence of breathlessness within the first ten days (OR 7.89; 95%CI 1.87–41.43; p = 0.008), an initial Ct value under 30 (OR 3.36; 95%CI 1.22–9.94; p = 0.022) as well as the occurrence of anosmia or ageusia within the first ten days (OR 3.01; 95%CI 1.10–8.84; p = 0.037) showed a statistically significant association with increased odds of illness duration over 90 days.

**Conclusion:**

About half of the HEs suffered from long lasting symptoms over 90 days after almost entirely mild acute COVID-19. Predictive factors could possibly be used for early treatment to prevent development of long-term symptoms after COVID-19 in future.

**Supplementary Information:**

The online version contains supplementary material available at 10.1186/s12879-023-08710-1.

## Introduction

Since the end of the year 2019, the emergence of severe acute respiratory syndrome coronavirus type 2 (SARS-CoV-2) has caused a global pandemic of coronavirus disease 2019 (COVID-19) infections [1, 2]. After the emergence of SARS-CoV-2, evidence increased that SARS-CoV-2 not only causes acute symptoms, but can also lead to long-lasting symptoms that can affect multiple organ systems. In addition to the burden of long-lasting COVID-19 symptoms on each individual affected, it has influence on the national economy, for example in the form of productivity loss and loss of gross value added due to reduced work capacity and sick leave [3].

After the first three waves of SARS-CoV-2 infections from early 2020 to springtime 2021 in Germany, a high number of infected hospital employees (HE) with long-lasting symptoms after predominantly mild acute COVID-19 courses could be observed at University Hospital Cologne-Merheim [4, 5]. In contrast to more frequently studied long-lasting symptoms after severe COVID-19 courses [6–8], there was a surprisingly high amount of long-lasting symptoms that also occurred after mild courses not requiring hospitalization. Since the vast majority of people infected with SARS-CoV-2 do not need to be hospitalized [9, 10], it is of great importance to also consider mild COVID-19 cases with regard to long-lasting COVID-19 symptoms.

Here, we had the opportunity to investigate the entire cohort of SARS-CoV-2 infected HEs. Although representativeness of hospital employees for the Cologne population can only be assumed but not proven, HEs were of the same age as the working population and could represent a rough estimate of the burden of long-lasting symptoms after SARS-CoV-2 infection. The aim of this study was to assess the frequency, duration and patterns of long-term COVID-19 symptoms in hospital employees and to identify risk factors for a prolonged course of non-severe COVID-19.

## Materials and methods

### Definition

Acute COVID-19 includes all signs and symptoms of COVID-19 lasting for up to 4 weeks [11].

The World Health Organization developed the following clinical case definition for post-COVID-19 syndrome: “Post COVID-19 condition occurs in individuals with a history of probable or confirmed SARS-CoV-2 infection, usually 3 months from the onset of COVID-19 with symptoms that last for at least 2 months and cannot be explained by an alternative diagnosis” [12].

The term ‘long COVID’ is frequently used and describes all signs and symptoms that persist or develop after acute COVID-19. It includes post-COVID-19 syndrome [11].

### Study setting

A systematic survey was conducted among SARS-CoV-2 positive hospital employees (HEs) of three medical centres in Cologne, Germany.

### Data collection

All HEs who had a positive SARS-CoV-2 polymerase chain reaction (PCR) test from the beginning of the pandemic in March 2020 until May 2021 were recorded by staff of the hospitals’ Institute of Hygiene. HEs were PCR tested by staff of the Institute of Hygiene before returning to work. If it was noticed that HEs were still affected by their SARS-CoV-2 infection, they were referred to the Occupational Health Service. HEs could also contact the Occupational Health Service independently. The Occupational Health Service of the three medical centres looked after HEs who had been affected by ongoing symptoms after SARS-CoV-2 infection and offered them medical support. COVID-19 infections due to occupational causes have been reported to the employers’ liability insurance association and rehabilitation measures were initiated.

For PCR testing, oro-nasopharyngeal swabs (eSwab® by Copan) or pharynx gargle samples were used [13]. In most cases, HEs were tested in our in-house COVID-19 test centres by trained medical staff. In some cases, for example outside the opening hours of our in-house test centres, HEs got their PCR test in the in-house emergency room or medical staff tested themselves independently on the wards. PCR analysis was performed in the diagnostic laboratories of the Institute of Pathology and Institute of Hygiene, which are affiliated with the hospital, and at the hospital’s laboratory medicine service provider. In our data analysis, only Ct values were used if the initial PCR was processed in one of our two in-house diagnostic laboratories [14].

All HEs with previous PCR confirmed SARS-CoV-2 infection were contacted by phone and asked about their willingness to participate in a systematic survey on possible long-term COVID-19 symptoms. HEs were given the option to cancel the survey at any time. The survey was conducted by staff of both the Occupational Health Service and the Institute of Hygiene and was carried out between June and October 2021. All HEs interviewed had at least a six-week interval from their first SARS-CoV-2 positive PCR result at the time of the interview. In preparation for the survey, a structured questionnaire was developed that included 24 different long-term COVID-19 symptoms that were frequently described in previously published studies about long-lasting COVID-19 symptoms [10, 15, 16](supplemental material). HEs were asked only about symptoms that occurred in connection with SARS-CoV-2 infection and were not asked to report symptoms that had previously existed. The self-assessed symptom severity (mild/medium/severe) and the respective time of symptom onset and end were asked. In addition, three different sources of infection were defined by physicians of the Institute of Hygiene and applied to each HE’s case. A definite nosocomial infection of HEs with SARS-CoV-2 was, by definition, present when there was a defined outbreak on a medical ward with exactly fitting time period. Contact with a COVID-19 case within the hospital with missing or inappropriate personal protective equipment within the last 14 days was also classified as a definite nosocomial infection. In the event of contact with a COVID-19 case within the hospital with appropriate personal protective equipment and lack of private contact to a COVID-19 case within the last 14 days, the infection was categorized as possible nosocomial infection. If there was contact with a COVID-19 case in the private setting or there was no contact situation within the healthcare facility within the last 14 days, this was categorized as a community-acquired COVID-19 infection.

### Data analysis

Descriptive analysis of frequency and duration of long-term COVID-19 symptoms were conducted. Analysis was performed using the Statistical Software R [17]. Using co-occurrence network analysis, the formation, patterns and chronology of COVID-19 symptom complexes after SARS-CoV-2 infection were assessed [18]. Survival-time analysis was conducted in order to assess the probability of respective symptom persistence using Kaplan-Meier estimator [19]. Likewise, analysis of symptom durations depending on professional group, age, initial Ct value and other influencing factors was carried out using Cox regression [20]. Possible risk factors for a protracted COVID-19 course with persistent COVID-19 symptoms over 90 days were identified using logistic regression [21]. As the Robert Koch Institute has decided to define a Ct value of 30 as the limit above which the infectivity decreases significantly regardless of the PCR method used, we used a dichotomized initial Ct (Ct value < 30 vs. Ct value ≥ 30) for the logistic regression [22, 23].

P values < 0.05 were considered statistically significant.

## Results

In total, 297 HEs tested positive for SARS-CoV-2 during our study period of which 221 HEs were interviewed for the survey. A number of 76 HEs were excluded because of survey refusal, survey drop out or incomplete survey. Mean age was 39.76 years (standard deviation (SD), 12.46; age range 17–64 years) and 158 HEs (71.5%) were female (Table 1). During the acute COVID-19 infection, five HEs (2.3%) received inpatient care, including two HEs (0.9%) who received intensive care treatment. At the time of SARS-CoV-2 PCR positivity, seven HEs (3.2%) had been vaccinated twice against COVID-19. Initial Ct values were available for 150 HEs (67.9%). Mean initial Ct value was 25.59 (SD 6.33; Ct value range 11–44; interquartile range (IQR) 8.75).

**Table 1:** Characteristics of investigated cohort of SARS-CoV-2 positive hospital employees

	Frequency		Symptom Duration, days		
	N	%	Median (IQR)	Mean	Min- Max
Total number	221	100.0	112 (210.0)	139.9	0- 491
**Demographics**					
Female	158	71.5	129 (209.8)	146.5	0- 491
Male	63	28.5	71 (189.0)	123.2	0- 452
**Age**					
16–30	70	31.7	70 (221.3)	116.6	0- 474
31–40	44	19.9	83.5 (184.5)	144.3	0- 482
41–50	55	24.9	145 (202.0)	137.0	0- 491
51–65	52	23.5	168 (175.5)	170.4	0- 482
**Professional group**					
Nursing staff	117	52.9	147 (214.0)	149.7	0- 491
Medical staff	24	10.9	50 (197.5)	128.5	0- 462
Others (e.g., administration, cleaning staff)	80	36.2	87.5 (194.3)	128.9	0- 474
**Working on a COVID ward**	59	27.0	182 (200.5)	162.5	0- 471
**Transmission type**					
Community acquired	114	51.6	57.5 (198.5)	117.5	0- 491
Definitely nosocomial	62	28.0	177 (168.5)	165.8	0- 482
Possibly nosocomial	45	20.4	183 (180.0)	160.8	0- 471

SD, standard deviation. Study population: hospital employees of three medical centres in Cologne, Germany, with SARS-CoV-2 positivity between March 2020 and May 2021

**Fig. 1:**
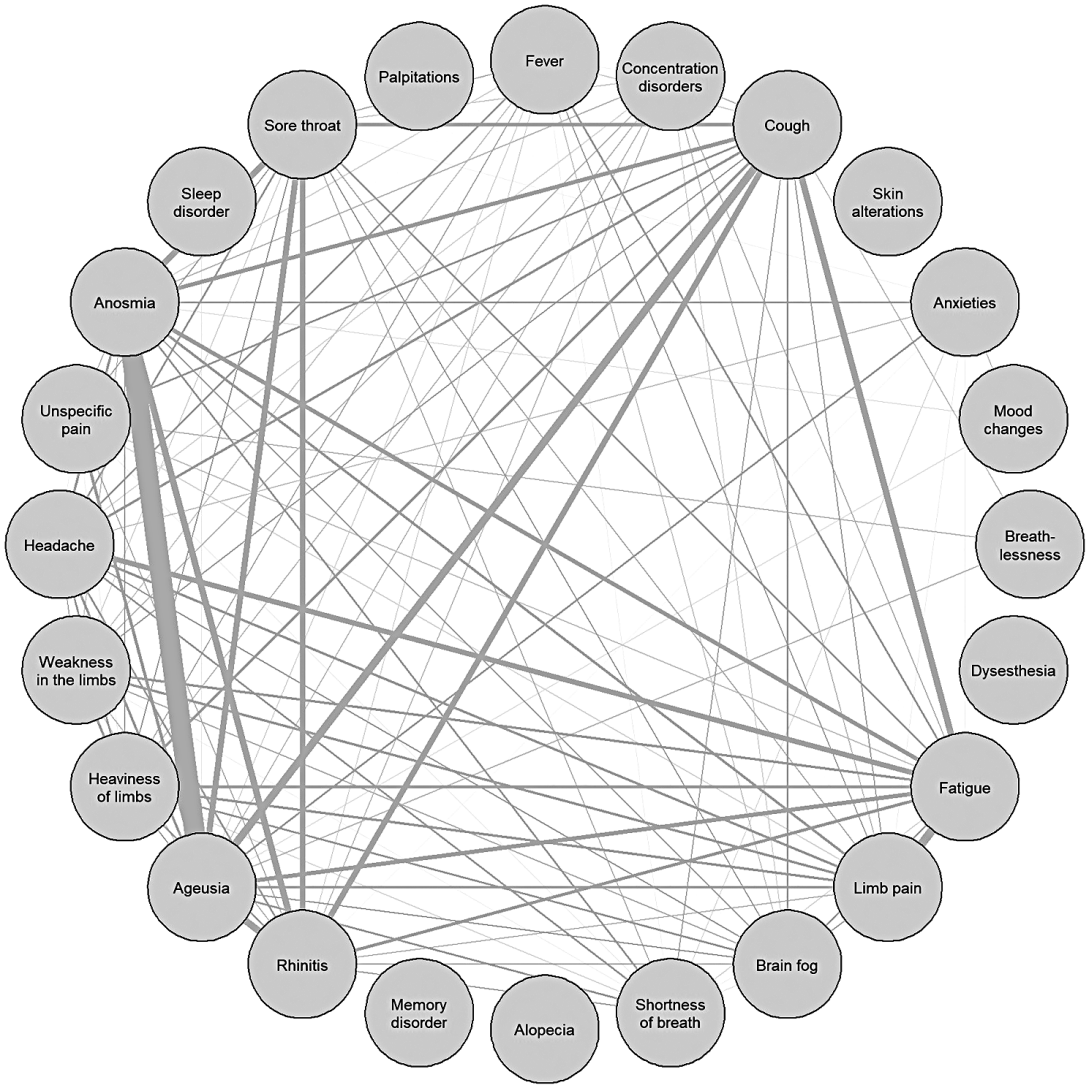
Co-occurrence network of symptoms. Symptom network includes symptoms only of hospital employees with symptom duration of maximum 28 days (N = 33). Each line shows the simultaneous occurrence of the respective symptoms. The thicker the line, the more often symptoms occurred simultaneously

**Fig. 2:**
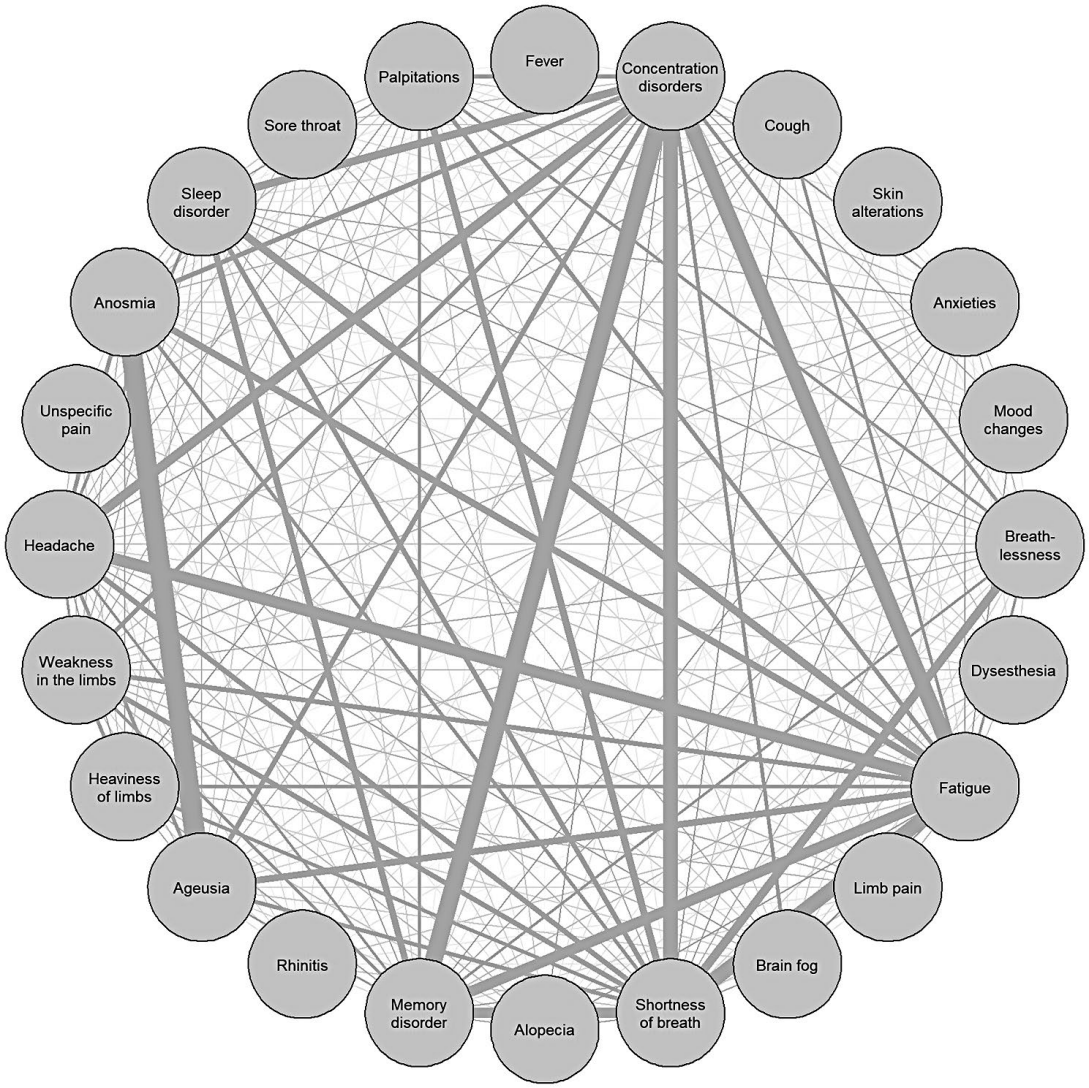
Co-occurrence network of symptoms. Symptom network includes symptoms only of hospital employees with symptom duration more than 28 days (N = 161). Each line shows the simultaneous occurrence of the respective symptoms. The thicker the line, the more often symptoms occurred simultaneously

Using co-occurrence network analysis, the formation of symptom complexes was demonstrated (Figs. 1 and 2). For 33 HEs who had a short symptom duration of less than 28 days, the most prominent combined symptoms were ageusia and anosmia (Fig. 1). With a symptom duration over 28 days (n = 161), a formation of additional network points of symptom complexes such as fatigue or concentration disorders, among others, could be seen. The most prominent symptom complex was the interrelated occurrence of shortness of breath, memory disorder, concentration disorders and fatigue (Fig. 2).

**Fig. 3:**
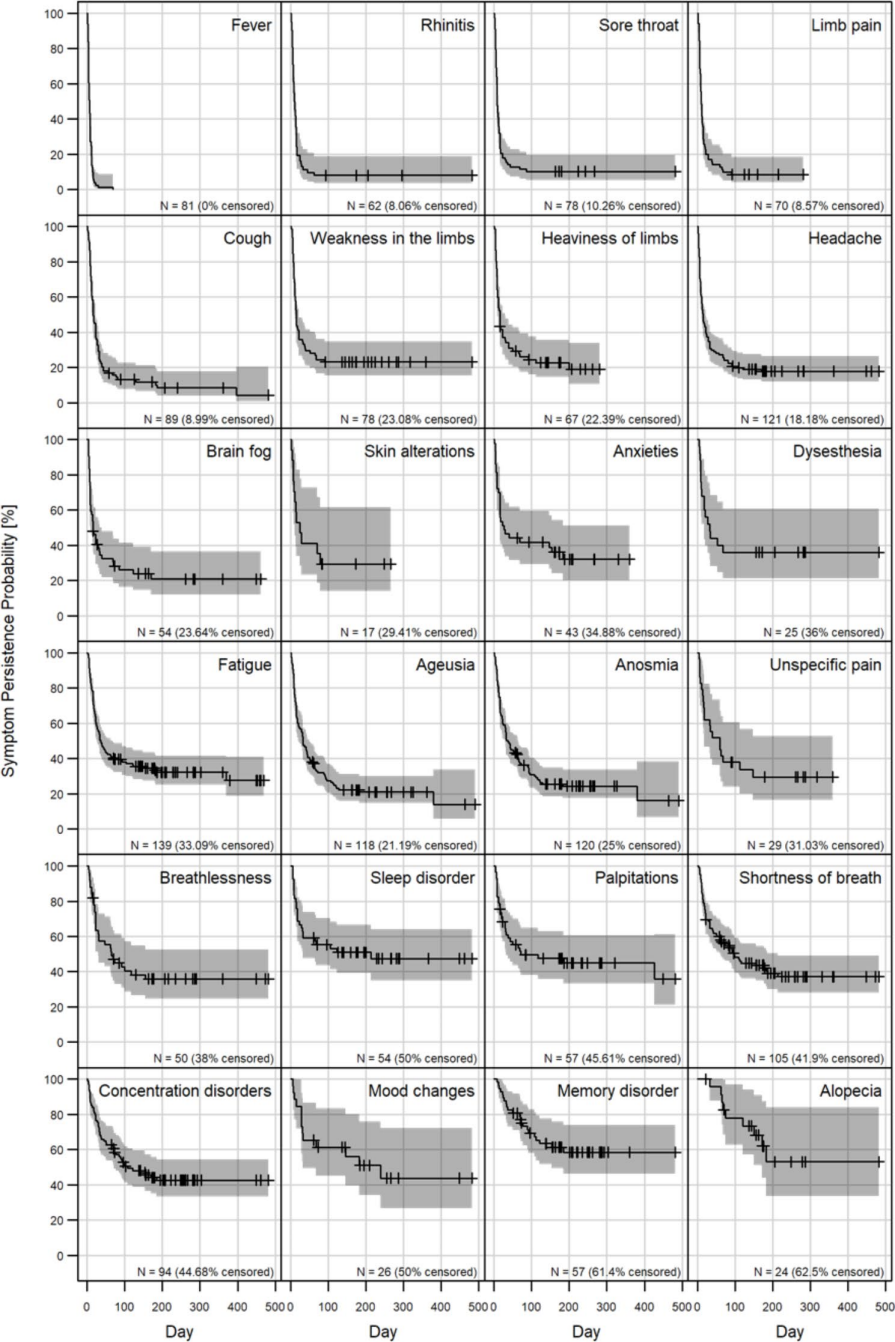
Kaplan-Meier curves of probability of symptom persistence. For each symptom, the number of hospital employees (N) who developed the respective symptom is stated. The percentage of censored data indicates how many hospital employees still had the respective symptom at the time of the survey. All graphs were arranged from top left to bottom right with increasing 28-days survival probability of the respective symptom

The probability of symptom persistence was plotted using Kaplan-Meier curves (Fig. 3). The symptom least likely to still be present after 90 days was fever (0.0% of n = 81), whereas alopecia was the symptom most likely to still be present after 90 days (78.0% of n = 24). For HEs who developed shortness of breath after SARS-CoV-2 detection (n = 105), the probability for persisting shortness of breath after 200 days was 39.0%. For HEs suffering from memory disorder (n = 57), the probability for persistence of memory disorder after 200 days was 58.4%.

Cox regression analysis showed that each one cycle higher initial Ct value significantly increased the chances of overcoming symptoms (odds ratio [OR] 1.05; 95% confidence interval (95%CI) 1.01–1.09; p = 0.019) (Table 3). The chances of being symptom-free for HEs who had a possibly nosocomial infection were significantly lower than for HEs with a community acquired SARS-CoV-2 infection (OR 0.48; 95%CI 0.24–0.98; p = 0.044). The Cox regression model also indicated that there was a trend to a higher chance of symptom resolving for medical staff than for nursing staff (OR 2.13; 95%CI 0.98–4.62; p = 0.055).

**Table 3:** Cox regression model for the event odds of becoming asymptomatic

	OR (95%CI)	SE	p value
Gender (female)	0.76 (0.44–1.31)	0.28	0.319
Age at the time of COVID disease	0.99 (0.97–1.01)	0.01	0.376
Professional group (Medical staff)	2.13 (0.98–4.62)	0.39	0.055
Professional group (Others)	1.02 (0.57–1.86)	0.30	0.938
Work on the COVID ward (yes)	0.89 (0.48–1.66)	0.32	0.711
Initial Ct value	1.05 (1.01–1.09)	0.02	**0.019**
Transmission type (Possibly nosocomial)	0.48 (0.24–0.98)	0.36	**0.044**
Transmission type (Definitely nosocomial)	0.59 (0.33–1.05)	0.30	0.074

OR; odds ratio. 95%CI; 95% confidence interval. SE; standard error. The event odds of becoming asymptomatic at day t are modelled. Only hospital employees with available initial Ct value were included (N = 150). For nominal scaled variables (gender, professional group, work on the COVID ward, transmission type), odds ratio refers to the reference characteristic (male gender, nursing staff, work on the non-COVID ward, community acquired). For interval scaled variables (age, Ct value), odds ratio refers to the characteristic that is one point/cycle higher in each case

To avoid misleading interpretation and since the modelled outcome is a positive event (asymptomatic), we refrain from hazard terminology in favor of odds

Logistic regression was used to identify factors that increased the probability of illness duration lasting more than 90 days (Table 4). The occurrence of breathlessness within the first ten days increased the probability of a prolonged illness duration over 90 days by a factor of OR = 7.89 (95%CI 1.87–41.43; p = 0.008), the presence of anosmia or ageusia increased the probability by a factor of OR = 3.01 (95%CI 1.10–8.84; p = 0.037). Initial Ct value < 30 (OR 3.36; 95%CI 1.22–9.94; p = 0.022) and a definitely nosocomial SARS-CoV-2 transmission (OR 3.05; 95%CI 1.03–9.67; p = 0.049) also showed a statistically significant correlation with increased odds of illness duration over 90 days.

**Table 4:** Odds for exceeding illness duration of 90 days based on logistic regression

	OR (95%CI)	SE	p value
Age (31–40)	0.97 (0.27–3.39)	0.64	0.960
Age (41–50)	1.96 (0.62–6.41)	0.59	0.256
Age (51–65)	3.03 (0.87–11.44)	0.65	0.089
Gender (female)	1.99 (0.69–6.07)	0.55	0.212
Professional group (Medical staff)	0.18 (0.03–0.95)	0.88	0.051
Professional group (Others)	0.52 (0.18–1.46)	0.53	0.222
Transmission type (Possibly nosocomial)	1.29 (0.41–4.09)	0.58	0.658
Transmission type (Definitely nosocomial)	3.05 (1.03–9.67)	0.57	**0.049**
Work on the COVID ward (yes)	1.24 (0.42–3.67)	0.55	0.700
Initial Ct value < 30	3.36 (1.22–9.94)	0.53	**0.022**
Rhinitis within the first 10 days	1.06 (0.34–3.30)	0.57	0.922
Concentration disorders within the first 10 days	2.28 (0.66–8.32)	0.64	0.197
Sore throat within the first 10 days	0.33 (0.11–0.98)	0.56	0.051
Fever within the first 10 days	2.81 (0.92–9.42)	0.59	0.079
Cough within the first 10 days	1.38 (0.48–4.05)	0.54	0.556
Breathlessness within the first 10 days	7.89 (1.87–41.43)	0.78	**0.008**
Shortness of breath within the first 10 days	1.09 (0.37–3.23)	0.55	0.878
Limb pain within the first 10 days	1.16 (0.34–4.09)	0.63	0.814
Unspecific pain within the first 10 days	2.17 (0.53–10.07)	0.74	0.297
Sleep disorder within the first 10 days	1.69 (0.41–7.22)	0.72	0.467
Brain fog within the first 10 days	0.70 (0.20–2.28)	0.61	0.554
Anxieties within the first 10 days	2.84 (0.52–18.54)	0.90	0.245
Mood changes within the first 10 days	0.78 (0.06–13.84)	1.37	0.856
Anosmia or Ageusia within the first 10 days	3.01 (1.10–8.84)	0.53	**0.037**
Heaviness of limbs or Weakness in the limbs within the first 10 days	0.63 (0.16–2.34)	0.68	0.488
Fatigue or Headache within the first 10 days	0.20 (0.05–0.68)	0.65	**0.014**

OR; odds ratio. 95%CI; 95% confidence interval. SE; standard error. Only hospital employees with available initial Ct value were included (N = 150). For nominal scaled variables, odds ratio refers to the reference characteristic (age ≤ 30, male gender, nursing staff, work on the non-COVID ward, community acquired, initial Ct value ≥ 30). Odds ratios were calculated for the presence of the listed symptoms within the first ten days after initial SARS-CoV-2 detection compared to the absence of the respective symptoms. Symptoms that occurred rarely were not considered. If two symptoms correlate strongly with each other, they are combined and the combined symptom is called observed as soon as one of the symptoms occurs

The accuracy data of logistic regression model were as followed: accuracy 0.73, cross-validation accuracy 0.61, sensitivity 0.79, cross-validation sensitivity 0.53, specificity 0.64, cross-validation specificity 0.50

## Discussion

In our cohort of SARS-CoV-2 positive tested and almost entirely still unvaccinated HEs during the first three waves (wild-type and alpha variant of SARS-CoV-2), a standardized interview revealed a high proportion of HEs with long lasting symptoms. Only 12% reported no symptoms at all, whilst 73% had symptoms longer than 28 days and 47% longer than 90 days. This goes in line with the work of Carvalho-Schneider et al., based on a prospective follow-up of 150 adults with noncritical COVID-19, that 68% of adults reported at least one lasting symptom at day 30 after symptom onset [24].

Comparison of different observational studies upon long lasting symptoms after non–severe SARS-CoV-2 infection revealed differences in the studied cohorts, the items of the interviews and the follow up. Whereas the first studies included patients who were asked for convalescent plasma donation or patients who were surveyed in the context of a quality management system [10, 15], here we included all employees of the Cologne Municipal Hospitals, representing the group of employees of working age in the healthcare sector in Cologne, Germany. Employees were not only tested for SARS-CoV-2 if symptoms were present, but also all direct contact persons were actively screened for SARS-CoV-2 for 14 days after the documented contact. Hence, we were able to also include HEs with an asymptomatic course. Interestingly, only 12% of the employees included in the survey had no symptoms at all. Compared to large register studies like Roessler et al. where disease diagnoses were recorded [25], in the present study we were able to capture symptoms that were not necessarily linked to a physician’s disease diagnosis. These symptoms may nevertheless be essential to understand the burden of post-COVID conditions [9].

In comparison to the work of Augustin et al. with a prevalence of 35% for six months lasting symptoms [10], in our cohort the prevalence of long-lasting symptoms after three months was at 47% of hospital employees, hence, being comparable.

A nationwide cross-sectional study in the Danish population with over 60.000 SARS-CoV-2 positive persons revealed significant risk differences for anosmia, fatigue/exhaustion, dyspnoea and reduced strengths in legs/arms compared to a SARS-CoV-2 negative control group [9]. Our data, which do not include control groups, are consistent with the findings of the Danish questionnaire study.

Our study showed the development of symptom complexes. Different symptom complexes following SARS-CoV-2 infection may be linked to different pathologies [26]. Thus, Zazhytska et al. reported that damaged cerebral cells with persistent viral loads in some individuals being possibly the cause for mental disorders, or a disruption of nuclear architecture may be a cause of anosmia [27]. Etter et al. showed signs of neurodegeneration associated to autoimmunity and peripheral immune signatures as possible causes for concentration and memory disorders or other central neurological symptoms [28]. Persistent infection or inflammation of heart cells may be the reason for palpitation and breathlessness [29, 30]. Furthermore, data of the electronic database of the US Department of Veterans Affairs indicated that individuals with COVID-19 had an increased risk for different cardiovascular diseases in comparison to controls [31].

In the logistic regression analysis, a statistically significant correlation was seen between definitely nosocomial SARS-CoV-2 transmission and increased odds of illness duration over 90 days. In the case of nosocomial acquisition, an increased infectious dose may have been present due to proximity to SARS-CoV-2 positive patients and initially limited resources for personal protective equipment. A correlation between infectious dose and severity of acute COVID-19 has been described [32, 33]. As pre-existing conditions and other factors were not taken into account and HEs had predominantly mild COVID-19 courses, the correlation of increased odds of illness duration over 90 days in the case of definitely nosocomial SARS-CoV-2 acquisition should nevertheless be evaluated with caution.

It must be mentioned that our study may be limited by a small sample size as well as a missing control group in our study design. Considering whether long-lasting symptoms were caused by SARS-CoV-2 infection or occurred incidentally, post-COVID symptoms need to be compared between SARS-CoV-2 positive and negative groups. The Danish cross-sectional study by Vedel Sørensen et al. as well as the findings of Roessler et al. confirm our data [9, 25]. Another limitation of our study is that we only asked about subjectively perceived symptoms. However, the work of Roessler et al. confirms our results and shows that also more severe conditions diagnosed by a trained physician are more common in the COVID-19 cohort than in the control cohort [25]. Cohen et al. detected an increased risk of 11% for SARS-CoV-2 infected patients over 65 years for persistent long-term symptoms [34]. In our questionnaire, we explicitly asked only about new onset symptoms. Nevertheless, we cannot rule out the possibility that some employees may have overstated their reported symptoms. As an additional limitation, it must be noted that no pre-existing conditions of the employees were taken into account and are therefore not included in the calculations. However, Subramanian et al. showed with their retrospective matched cohort study, in which they used data from a UK-based primary care database and included comorbidities, that people with a confirmed SARS-CoV-2 infection had an increased risk of, for example, shortness of breath or anosmia after more than 12 weeks. As in our study, SARS-CoV-2 infections from the period when wild-type and alpha variants dominated were analysed [35]. We identified several predictors for long-lasting COVID-19 symptoms. As medical conditions other than those included in our analysis could not be considered, we cannot rule out the existence of other important predictors of long-lasting COVID-19. As a further limitation, it must also be mentioned that the professional group “Others” is very heterogeneous and that both non-patient related personnel such as administrative staff as well as patient-related personnel such as cleaning staff are combined in one group. Especially with regard to the transmission type of nosocomial acquisition and the correlation to symptom duration, the heterogeneity of this group must be taken into account.

Based on symptom complex analyses, it was shown that employees with a short symptom duration of less than 28 days experienced mainly cold symptoms and loss of smell and taste, which subsided soon after the acute infection. With a longer symptom duration, symptoms such as fatigue or concentration disorders became increasingly apparent. These long-lasting symptoms often occur in combination and represent a high burden for the affected individuals. It also has a major socioeconomic impact. The socioeconomic impact of long lasting symptoms after a SARS-CoV-2 infection was early described by the surrogate sick leave of working people in Sweden. Westerlind et al. found out in their study that the median leave time from work was 35 days and 9% were absent from work for at least four months due to COVID-19 [16].

For both medical and socioeconomic reasons, predictors for long lasting symptoms are essential. Predictors were first identified by the Swedish study investigating sick leaves, which were older age and sick leaves within the year prior to infection in a first rough analysis [16]. Through the identification of risk factors for long-lasting COVID-19 course, individuals who are at increased risk can be selected and medically treated at an early stage [8]. A recently published study from Xie et al. examined the association of antiviral treatment during acute COVID-19 and the risk of post-COVID-19 syndrome. Using health care databases of the US Department of Veterans Affairs, this cohort study found out that in people with at least one risk factor for a severe course of disease who had been treated with nirmatrelvir in the early stages of acute COVID-19, the risk of post-acute sequelae such as cardiovascular and neurologic system impairment, fatigue and malaise, or shortness of breath was reduced [36]. It is possible that the early predictors we identified may help to target antiviral therapy in future.

Kuodi et al. examined the effectiveness of COVID-19 vaccines against long-term COVID-19 symptoms and showed, that vaccination with at least two doses of COVID-19 vaccine reduced the odds for post-COVID symptoms substantially and even bringing it to the same baseline like individuals reporting no previous SARS-CoV-2 infection [37]. Therefore, the importance of full vaccination against COVID-19 must be emphasized also with regard to the prevention of long-lasting COVID-19 symptoms.

## Conclusion

A large number of HEs reported long-lasting symptoms after predominantly mild acute COVID-19 infections. The most common symptoms were fatigue, headache, anosmia and ageusia. We identified predictive factors for long-lasting COVID-19 symptoms such as the occurrence of breathlessness within the first ten days of acute COVID-19. In the future, predictive factors and risk factors could possibly be used to prevent the occurrence of long-term symptoms after COVID-19 with the help of early treatment.

### Electronic supplementary material

Below is the link to the electronic supplementary material.

Supplementary Material 1

Supplementary Material 2

## Data Availability

The datasets used and generated in this study are included in this published article and its supplementary information files.
