# Supplementary material for: Long-term symptoms after SARS-CoV-2 infection in a cohort of hospital employees: duration and predictive factors
Source: BMC Infect Dis. 2024 Jan 23;24:119. doi: 10.1186/s12879-023-08710-1 (PMC10807182; doi:10.1186/s12879-023-08710-1)
Supplement: Supplementary file 1 — Supplementary Material 1 [file 12879_2023_8710_MOESM1_ESM.pdf]

Supplemental Material

Questionnaire For Hospital Employees On Persistent COVID-19 symptoms

Patient/Hospital Employee (last name, first name, date of birth):____

Date of survey:___________Who has surveyed?_____________

Has the survey been agreed to? Yes/No Was the survey broken off? Yes/No

How was the course of your SARS-CoV-2 infection?

____(free text, use back page if necessary)

At what point did you feel fully fit again? ____________________(date)

What do/did you suffer from (beyond preexisting symptoms prior your SARS-CoV-2 infection)?

1. Fatigue no/yes: from__________until__________manifestation:____
2. Sleep disorder no/yes: from__________until__________manifestation:____
3. Anosmia no/yes: from__________until__________manifestation:____
4. Ageusia no/yes: from__________until__________manifestation:____
5. Headache no/yes: from__________until__________manifestation:____
6. Dysesthesia no/yes: from__________until__________manifestation:____
7. Brain fog no/yes: from__________until__________manifestation:____
8. Concentration disorders no/yes: from__________until__________manifestation:____
9. Memory disorder no/yes: from__________until__________manifestation:____
10. Anxieties no/yes: from__________until__________manifestation:____
11. Mood changes no/yes: from__________until__________manifestation:____
12. Limb pain no/yes: from__________until__________manifestation:____

13. Heaviness of limbs no/yes: from____________until____________manifestation:____

14. Unspecific pain no/yes: from____________until____________manifestation:____

15. Weakness in the limbs no/yes: from____________until____________manifestation:____

16. Sore throat no/yes: from____________until____________manifestation:____

17. Fever no/yes: from____________until____________manifestation:____

18. Alopecia no/yes: from____________until____________manifestation:____

19. Rhinitis no/yes: from____________until____________manifestation:____

20. Palpitations no/yes: from____________until____________manifestation:____

21. Breathlessness no/yes: from____________until____________manifestation:____

22. Shortness of breath no/yes: from____________until____________manifestation:_________________________ How many stairs can you walk? ______

23. Cough no/yes: from____________until____________manifestation:____

24. Skin alterations no/yes: from____________until____________manifestation:__________________ What kind?______________________________

25. Others? no/yes: from____________until____________manifestation:__________________ What kind?______________________________

Institute of Hygiene/Occupational Health Service. Questionnaire developed based on previously published studies[1-3]

Literature sources:

1. Augustin M, Schommers P, Stecher M, et al. Post-COVID syndrome in non-hospitalised patients with COVID-19: a longitudinal prospective cohort study. *The Lancet regional health Europe* 2021; **6**: 100122.
2. Jones R, Davis A, Stanley B, et al. Risk Predictors and Symptom Features of Long COVID Within a Broad Primary Care Patient Population Including Both Tested and Untested Patients. *Pragmatic and observational research* 2021; **12**: 93-104.
3. Westerlind E, Palstam A, Sunnerhagen KS, Persson HC. Patterns and predictors of sick leave after Covid-19 and long Covid in a national Swedish cohort. *BMC public health* 2021; **21**(1): 1023.
